# Supplementary material for: Enumeration and Characterization of Circulating Tumor Cells in Patients with Hepatocellular Carcinoma Undergoing Transarterial Chemoembolization
Source: Int J Mol Sci. 2023 Jan 29;24(3):2558. doi: 10.3390/ijms24032558 (PMC9916725; doi:10.3390/ijms24032558)
Supplement: Supplementary file 1 [file ijms-24-02558-s001.zip › ijms-2142431-supplementary.pdf]

International Journal of *Molecular Sciences*

MDPI

Supplementary Table S1. Relationship between clinicopathological characteristics of patients and $CD45^+$ CK^+ cell count. Individual count of double positive cells and the sum of $CD45^-$ CK^+ CTCs and double positive cells are shown.

Variable	Patients n (%)	Cell count n=37; 100.0%)			
		**$CD45^+$ CK^+ cells**		**CTCs and $CD45^+$ CK^+ cells**	
		**Median (IQR)**	**p**	**Median (IQR)**	**p**
Age (years)					
- <65	18 (48.6%)	27.0 (16.-90.5)		58.3 (37.5-186.3)	
- ≥65	19 (51.4%)	30.0 (15.0-54.0)	0.796	68.0 (44.0-116.0)	0.574
Sex (male)	34 (91.9%)	29.0 (17.5-60.3)	0.242	68.0 (44.8-138.3)	0.559
Etiology					
- Hepatitis C (yes)	14(37.8%)	31.0 (15.5-134.0)	0.364	57.0 (44.8-398.5)	0.616
- Hepatitis B (yes)	5 (13.5%)	48.0 (18.5-76.0)	0.437	116.0 (50.0-138.5)	0.625
- Alcohol (yes)	27 (73.0%)	26.0 (18.0-58.0)	0.932	68.0 (44.0-139.0)	0.918
- NAFLD (yes)	4 (10.8%)	16.0 (4.0-85.0)	0.240	52.0 (26.3-224.8)	0.509
Child-Pugh score					
- A	32 (86.5%)	29.0 (16.5-57.0)		68.0 (44.3-138.0)	
- B	5 (13.5%)	23.0 (9.0-121.0)	0.706	46.0 (27.0-1017.5)	0.982
MELD score					
- <9	14 (37.8%)	28.5 (16.0-61.8)		57.0 (43.03-138.3)	
- ≥9	23 (62.2%)	28.0 (15.0-60.0)	0.900	68.0 (37.0-151.0)	0.802
BCLC stage					
- 0-A	26 (70.3%)	30.0 (15.8-76.0)		69.0 (43.3-143.3)	
- B-D	11 (29.7%)	25.0 (18.0-48.0)	0.572	64.0 (34.0-138.0)	0.702
Portal Hypertension (yes)	31 (83.8%)	28.0 (16.0-60.0)	0.695	68.0 (44.0-139.0)	0.821
Number of nodules					
- Single nodule	18 (48.6%)	24.5 (15.5-48.3)		68.0 (37.8-141.3)	
- Multinodular	19 (51.4%)	32.0 (16.0-67.0)	0.638	64.0 (44.0-139.0)	0.761
Main nodule diameter (cm)					
- <3	17 (45.9%)	54.0 (21.0-101.5)		116.0 (48.5-272.5)	
- ≥3	20 (54.1%)	22.5 (14.5-32.0)	0.024	47.0 (36.3-73.3)	0.038
Total tumor diameter (cm)					
- <5	24 (64.9%)	29.0 (16.5-71.5)		69.0 (45.3-151.5)	
- ≥5	13 (35.1%)	25.0 (13.5-51.0)	0.316	53.0 (33.0-138.5)	0.408
Number of treated nodules					
- Single nodule	22 (59.5%)	24.0 (16.0-48.3)		66.0 (43.3-141.3)	
- >1 nodule	15 (40.5%)	36.0 (15.0-67.0)	0.536	107.0 (34.0-139.0)	0.901
AFP (ng/ml)					
- Normal (<9)	17 (45.9%)	32.0 (15.0-66.5)	0.779	70.0 (37.0-153.5)	0.499
- Abnormal (≥9)	18 (48.6%)	26.5 (15.8-55.0)		58.5 (44.8-120.0)	
- missing	2 (5.4%)				

AST (U/L)					
- Normal (<35)	18 (48.6%)	25.5 (14.0-61.8)		66.0 (35.5-152.3)	
- Abnormal (≥35)	19 (51.4%)	32.0 (16.0-58.0)	0.638	68.0 (44.0-132.0)	0.939
ALT (U/L)					
- Normal (<27)	19 (51.4%)	25.0 (18.0-48.0)		53.0 (36.0-138.0)	
- Abnormal (≥27)	18 (48.6%)	37.0 (15.8-76.0)	0.475	72.5 (46.5-143.3)	0.386
GGT (U/L)					
- Normal (<75)	17 (45.9%)	25.0 (17.0-35.0)		50.0 (35.5-144.5)	
- Abnormal (≥75)	20 (54.1%)	40.5 (15.3-65.3)	0.532	91.0 (45.5-138.8)	0.385
Tumor vascularity					
- Hypervascularity	23 (62.2%)	30.0 (18.0-67.0)		75.0 (47.0-156.0)	
- Medium/Hypo-vascularity	14 (37.8%)	23.0 (13.8-50.5)	0.372	46.5 (31.5-119.3)	0.060
